# Magnetic resonance metrics to evaluate the effect of therapy in amyotrophic lateral sclerosis: the experience with edaravone

**DOI:** 10.1007/s00415-021-10495-9

**Published:** 2021-03-02

**Authors:** Eugenio Distaso, Giammarco Milella, Domenico Maria Mezzapesa, Alessandro Introna, Eustachio D’Errico, Angela Fraddosio, Stefano Zoccolella, Franca Dicuonzo, Isabella Laura Simone

**Affiliations:** 1 Neurology Unit, Department of Basic Medical Sciences, Neurosciences and Sense Organs, University of Bari “Aldo Moro”, Piazza Giulio Cesare 11, 70124 Bari, Italy; 2 ASL Bari, San Paolo Hospital, Milano, Italy; 3 Neuroradiology Unit, Department of Basic Medical Sciences, Neurosciences and Sense Organs, University of Bari “Aldo Moro”, Piazza Giulio Cesare 11, 70100 Bari, Italy

**Keywords:** ALS, MRI, Edaravone, Cortical thickness, Fractional anisotropy, Longitudinal study

## Abstract

**Background:**

Edaravone was approved as a new treatment for amyotrophic lateral sclerosis (ALS), although there are different opinions on its effectiveness. Magnetic resonance (MRI) measures appear promising as diagnostic and prognostic indicators of disease. However, published studies on MRI using to monitor treatment efficacy in ALS are lacking.

**Purpose:**

The objective of this study was to investigate changes in brain MRI measures in patients treated with edaravone.

**Methods:**

Thirteen ALS patients assuming edaravone (ALS-EDA) underwent MRI at baseline (T0) and after 6 months (T6) to measure cortical thickness (CT) and fractional anisotropy (FA) of white matter (WM) tracts. MRI data of ALS-EDA were compared at T0 with those of 12 control subjects (CS), and at T6 with those of 11 ALS patients assuming only riluzole (ALS-RIL), extracted from our ALS cohort using a propensity-score-matching. A longitudinal MRI analysis was performed in ALS-EDA between T6 and T0.

**Results:**

At T0**,** ALS-EDA showed a cortical widespread thinning in both hemispheres, particularly in the bilateral precentral gyrus, and a reduction of FA in bilateral corticospinal tracts, in comparison to CS. Thinning in bilateral precentral cortex and significant widespread reduction of FA in several WM tracts were observed in ALS-EDA at T6 compared to T0. At T6, no significant differences in MRI measures of ALS-EDA versus ALS-RIL were found.

**Conclusions:**

Patients treated with edaravone showed progression of damage in the motor cortex and several WM tracts, at a six-month follow-up. Moreover, this study showed no evidence of a difference between edaravone and riluzole.

## Background

Amyotrophic lateral sclerosis (ALS) is a neurodegenerative disease causing weakness and wasting of voluntary muscle, associated in about 50% of cases with cognitive impairment [1]. Although the pathogenic mechanism is not yet understood, oxidative stress caused by free radicals seems to be an essential factor involved in motor neuron degeneration and in the progression of the disease [2].

Edaravone is a free-radical scavenger of peroxyl radical that could potentially reduce the postulated oxidative stress in ALS. For this reason, edaravone has been proposed, approved and licensed as the first new treatment for ALS after riluzole. The first phase 3 study did not show significant differences in the ALSFRS-r score between patients receiving edaravone and placebo; however, post hoc analyses identified a subpopulation in which edaravone showed efficacy [3, 4]. To date, there are several reports on the clinical efficacy and safety of edaravone, with promising but discordant results [5–8]. More recently, no differences in ALS patients treated and not treated with edaravone in terms of disease progression and respiratory function has been reported in a multicenter Italian real-life study [9].

Magnetic resonance imaging (MRI) plays a role in the diagnostic work-up of ALS and currently, its importance is further growing up by the advent of neuroimaging putative biomarkers [10]. Newer MRI techniques appear promising as diagnostic and prognostic indicators of disease. In particular, surface-based cortical thickness measures, voxel-based morphometry for regional grey matter volume variations and diffusion tensor imaging (DTI) metrics of white matter tracts have gained wide consensus [11]. To our knowledge, there are no published studies on MRI measures as an indicator of the effectiveness of drugs in ALS.

The objective of this study was to investigate changes in brain MRI measures in patients treated with edaravone.

## Materials and methods

### Population

Three groups of subjects were studied: (a) ALS patients taking edaravone in addition to riluzole (13 patients; ALS-EDA); (b) control subjects (12 subjects; CS); (c) ALS patients taking only riluzole (11 patients; ALS-RIL). All subjects included in the study underwent MRI. ALS patients were recruited at the tertiary center of motor neuron diseases of our Department.

Thirteen patients, who met the criteria of the Italian Drug Agency for edaravone administration, were enrolled in the first group (ALS-EDA). Inclusion criteria were clinically “probable” or “definite” ALS according to revised El Escorial Criteria [12], age > 18 years, disease duration less than 2 years, forced vital capacity (FVC) ≥ 80% predicted normal value for gender-height-age in a seated position at the screening visit, a subscore ≥ 2 in all items of ALSFRS-r score and a decrease in the ALSFRS-r score of 1–4 during a 12-week observation period between the screening and the baseline. Exclusion criteria were concomitant significant neurological or neurodegenerative diseases, concomitant significant diseases in other systems or organs, creatine clearance lower than 50 mL/min, pregnant or breastfeeding women, and patients who do not understand or provide informed consent. At inclusion, all patients had to take riluzole 100 mg/day for at least one month. These patients underwent clinical evaluations by a neurologist of the ALS team and MRI investigations at baseline (T0, in 13 patients) and after 6 months (T6, in 11 patients). The following clinical data were collected: site of onset (bulbar or spinal), sex, age, disease duration from symptoms onset, ALSFRS-r, progression rate [13]. Furthermore, each patient has been evaluated for a degree of UMN burden, using Penn Upper Motor Neuron score [14], stratifying the study population using 50% of the total score as cut-off. At follow-up, two patients dropped out, one due to death and one because of inability to perform magnetic resonance imaging for severe orthopnea.

Controls (CS) were age and sex-matched subjects to edaravone group, and consisted of 12 subjects not affected by neurodegenerative diseases, family history of ALS, and with normal brain MRI, that was performed in the diagnostic work-up of their disease.

The third group included 11 definite or probable ALS patients, extracted from a cohort of 40 patients of our Apulia ALS registry, all treated with riluzole (ALS-RIL), who underwent the same MRI protocol of ALS-EDA group. The comparative MRI findings at T6 of ALS-EDA versus ALS-RIL was assessed in propensity score-matched groups. The aforementioned clinical data were collected for ALS-RIL patients.

All ALS patients underwent physiotherapy from the diagnosis of the disease at our tertiary centre; however, the considering the lack of a standardized protocol for this treatment, this latter was not included as variable influencing MRI outcome.

Written informed consent was obtained from each participant according to the Declaration of Helsinki and the study was approved by the Interregional Independent Ethical Committee of “Azienda Ospedaliero- Universitaria” of Bari-Italy.

### MRI acquisitions

All participants underwent MRI on a Philips MR system 1.5 T scanner. CS and ALS-RIL groups underwent the same brain MRI protocol once, while ALS-EDA patients twice, at T0 and T6.

Routine T1, T2 weighted sequences and fluid-attenuated inversion recovery (FLAIR) were performed to exclude unrelated abnormalities. 3D-structural MRI was acquired using a T1-weighted MP-RAGE (magnetization-prepared rapid acquisition with gradient echo) sequence (TR/TE/flip angle: 25.00 ms/4.60 ms/30.00 degree; Field Of View [FOV]:240 mm; matrix 256 × 256, voxel size 0.93 × 0.93x1.0 mm^3^). For Diffusion Tensor Imaging (DTI) analyses, we used a spin echo DTI sequence: 1.75 × 1.75 × 2.5 mm^3^ acquiring voxel size, reconstructed matrix 128 × 128; 60 slices; TE/TR 77.791/3461 ms; flip angle 90°; a DTI diffusion scheme was used, and a total of 16 diffusion sampling directions were acquired. The b value was 800 s/mm2. The in-plane resolution was 1.75 mm. The slice thickness was 2.5 mm.

### Cortical thickness (CT) analysis

FreeSurfer software v.7.1 was employed to assess CT. Processing steps included correction for magnetic field inhomogeneity, alignment to a specific atlas [15], skull removal and segmentation of voxels into grey matter (GM), white matter (WM), and cerebrospinal fluid (CSF). CT was then calculated based on the shortest distance of two surfaces: the interface between GM and WM and the pial surface. A Gaussian filter of 10 mm full width at half maximum was used for smoothing in all analyses.

We employed the longitudinal FreeSurfer pipeline to evaluate 6-month CT changes. The software co-registers the two-time points scan for each subject using a robust and inverse consistent registration algorithm to create an unbiased subject-specific template [16, 17]. Then, several steps in the longitudinal processing stream are initialised from a subject-specific template. This approach has been shown to increase reliability and statistical power [18].

### Cerebral WM analysis

The DTI datasets were processed with the FMRIB Software Library v6.0 (FSL) software package. Pre-processing included denoising, removing Gibb’s ringing artefacts, eddy currents, and motion correction. Afterwards, a diffusion tensor model was fitted at each voxel, generating maps of fractional anisotropy (FA), performed with the tract based spatial statistics (TBSS) algorithm, as described elsewhere [19]. Our longitudinal TBSS pipeline follows the steps proposed by Menke et al. [20]. FA maps of each patient in native space were linearly registered into halfway space and averaged. To accomplish that, both images were linearly registered to each other, and then the transformation matrix into halfway space was calculated. This transformation was applied in both images, thus resulting in an average image. Afterwards, we ran the standard TBSS protocol**.**

### Statistical analysis

In descriptive clinical analyses, continuous variables were summarised as mean ± SD as median and range, and categorical variables were expressed as relative frequencies. Mann–Whitney and Fisher’s exact test were assessed for comparisons demographic and clinical features between ALS-EDA at T0 and CS groups, between ALS-EDA at T6 and ALS-RIL groups.

(a) The first step of the study included a cross-sectional analysis of MRI findings in ALS-EDA at T0 versus CS. Furthermore to define the effect of edaravone as addition therapy to riluzole on MRI patterns, ALS-EDA at T6 were compared to ALS-RIL, this latter including patients taking riluzole for the same duration of ALS-EDA. Then, to allow for an unbiased comparison, ALS-RIL were selected from a pool of 40 ALS patients using a propensity score-matched on a one-to-one basis, at the time of MRI concerning riluzole treatment duration. One-to-one matching was performed based on nearest neighbour matching within a calliper of a width of 0.2 standard deviations [21]. Site of onset (bulbar or spinal), sex, age, disease duration from symptoms onset, ALSFRS-r, progression rate [13], and predominant upper or lower motor neuron were used as covariates. Finally, 11 ALS-RIL patients were retained for the comparison with 11 ALS-EDA patients at T6.

(b) The second step of the study included a longitudinal analysis comparing paired MRI findings in ALS-EDA at T6 vs T0.

### Cross-sectional MRI analysis

The group differences of CT between ALS-EDA at T0 and CS and between ALS-EDA at T6 and ALS-RIL were explored with two types of analysis: a vertex-based analysis and a region of interest (ROI)-based one. The whole-brain vertex-wise analysis is a point-by-point group comparison of thickness across the cortical surface, without any a priori hypothesis, starting with the average images of each group. This statistical analysis was performed using Qdec (Query, Design, Estimate and Contrast), a module of Freesurfer, developed to design and execute surface analysis using age and gender as covariates. To correct for multiple comparisons, we performed Monte-Carlo cluster-based simulation with 10.000 permutations and we searched for significant clusters with p value level 0.05[22]. Areas showing significant cortical thinning were superimposed on the template. The vertex-analyses were supplemented by a ROI analysis, using the mean values of CT in the primary motor area (bilateral precentral gyrus).

WM analysis was employed on FA parameters using a two-sample t-test, with cluster-based correction for multiple comparisons (*p *value = 0.05) using Threshold Free Cluster-Enhancement (TFCE) correction [23]. Johns Hopkins white matter DTI-based atlas (available in the FSL software) was employed to identify WM tracts with abnormal findings and to extract their FA mean values. Between groups comparison was assessed using the non-parametric Mann–Whitney *U* test.

### Longitudinal MRI analysis

The longitudinal analysis compared T6 vs T0 paired MRI findings in ALS-EDA patients. Because all subjects have the same number of time points, symmetrized percent change (SPC), which is a dimensionless measure of change, was computed at each vertex for each participant to assess percent change [18]. SPC is defined according to the subsequent formula: SPC = 100 × (V2 − V1) 0.5 × (V1 + V2), where V1 is the vertex-wise brain measure at T0 and V2 is the measure at 6-month follow-up. SPC maps of CT were computed for each subject. Finally, one-sample group mean-test was performed to test if the symmetrized percent change in our sample is different from zero. Results were corrected for multiple comparisons with a two-tailed permutation simulation [24], using a cluster-wise forming threshold of *p* < 0.05 and 10 000 random permutations. Maps were visualized by overlaying significant clusters on top of the cortical surface in the visualization tool Freeview. Longitudinal TBSS protocol was performed using a paired two-sample t-test with TFCE [23] correction (alpha = 0.05) and the results were projected to FA MNI 152 template. We then applied a mask on the results to extract bilateral corticospinal tract (CST) mean FA. Within group comparisons were performed using a Wilcoxon signed-rank test.

### Sample size calculation

A minimum sample size was calculated to assess the effect of edaravone on CT of precentral gyrus and FA of CST. A free software available at http://hedwig.mgh.harvard.edu/sample_size/size.html was used for sample size calculation. To detect a mean difference of 0.25 mm in CT of precentral gyrus, as previously indicated in literature [25], with a standard deviation of 0.13 [26], a total of 12 patients had to be entered in this two treatment parallel-design study, considering a power of 80%, a type I error rate of 0.05 and two-sided analyses. Regarding the effect of edaravone on FA of CST, to our knowledge, there are not cross-sectional studies evaluating the minimal detectable change of FA of CST in two compared groups of ALS patients. Considering that the propensity score-matching method limited our analysis to 22 MRI examination, the minimal difference in FA of CST detectable by our study was 0.038, with a 0.03 standard deviation of CST [27], a power of 80%, a type I error rate of 0.05 and two-sided analyses.

## Results

Clinical and demographic features of ALS-EDA at T0, CS, ALS-EDA at T6 and matched ALS-RIL are summarized in Table 1

**Table 1:** Demographic and clinical features in study populations

	ALS-EDA at T0 n. 13	CS n. 12	ALS-EDA at T6 n.11	ALS-RIL n.11
Age (mean ± SD) (median; range) (years)	50.23 ± 7.32 49; 40–61	54.17 ± 8.55 53 ; 40–67	49.73 ± 7.04 49; 40–61	55.64 ± 7.42 58; 45–66
Sex (male/female)	7/6	7/5	7/4	6/5
Site of symptom onset (bulbar/spinal)	4/9	N/A	3/8	4/7
Disease onset in months at MRI (mean ± SD) (median; range)	13 ± 4 13; 8–23	N/A	19 ± 5 18; 12–28	16 ± 8 15; 5–32
Progression Rate at MRI (mean ± SD) (median; range)	0.44 ± 0.21 0.44; 0.13–0.92	N/A	0.79 ± 0.37 0.73; 0.30–1.50	0.56 ± 0.36 0.54; 0.17–1.44
UMN/LMN burden	10/3	N/A	8/3	7/4

Mann–Whitney *U* test: p = ns; Fisher’s exact test p = ns

*ALS-EDA* patients taking edaravone in add on to riluzole, *ALS-RIL* patients taking riluzole, *CS* control subjects, *MRI* magnetic resonance imaging, *UMN/LMN* burden: patients with prevalent upper or lower motor neuron burden at the time of MRI using 50% of the total score of Penn Upper Motor Neuron Scale as cut-off

### Cross-sectional MRI analysis

**Fig. 1:**
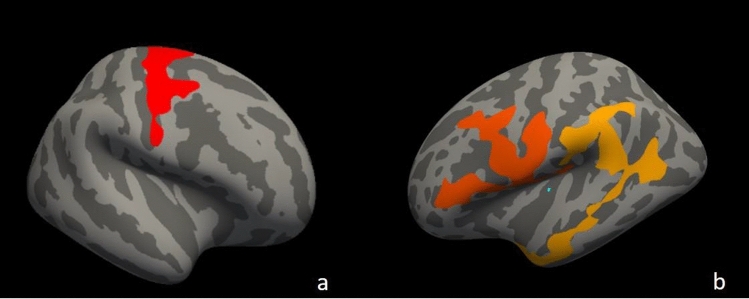
Vertex-wise analysis of CT in ALS-EDA patients related to CS. ALS-EDA revealed cortical thinning in the right precentral and paracentral cortex (**a**) and in the left precentral, pericalcarine and inferior-parietal cortex (**b**). The coloured areas represented the significance maps after Monte Carlo cluster-wise correction

**Fig. 2:**
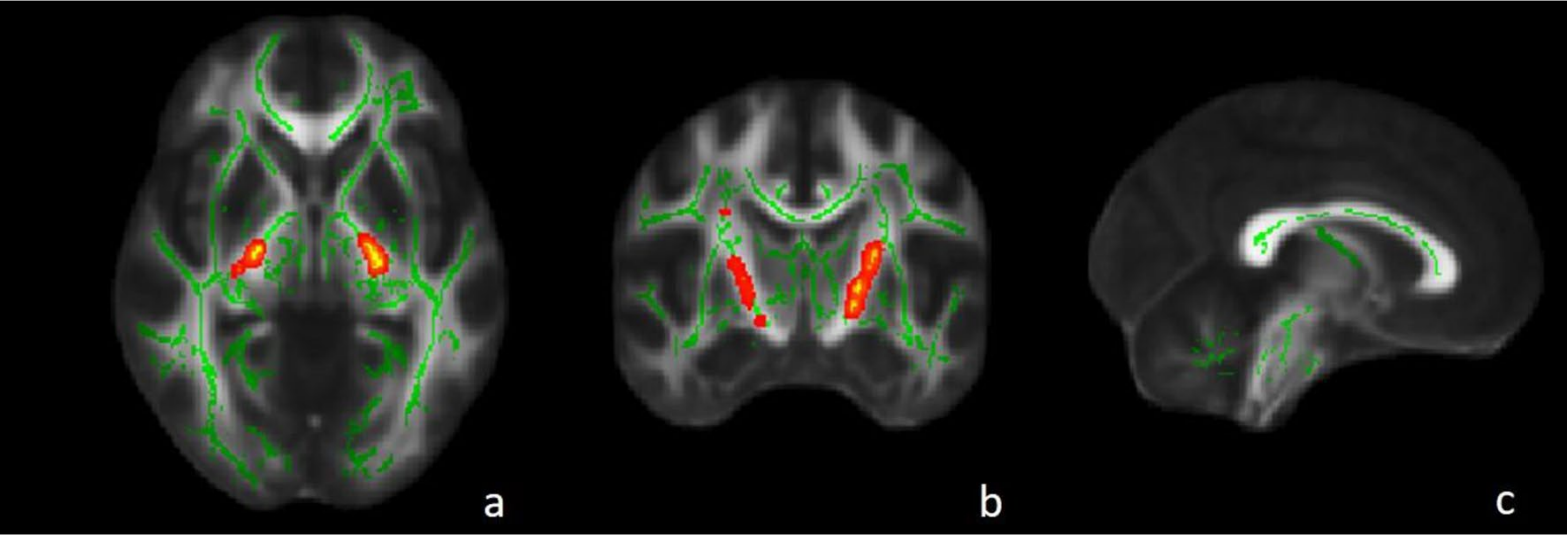
TBSS analysis of ALS-EDA patients at T0 compared to CS. Areas with reduced FA are shown in yellow–red in bilateral CST from the cortex to the cerebral peduncle in ALS-EDA vs CS. Regions of significant differences are overlaid on axial (**a**), coronal (**b**) and sagittal (**c**) of the MNI152 1 mm template

ALS-EDA at T0 showed a widespread cortical thinning in the left hemisphere, in particular in the precentral, pericalcarine and inferior-parietal cortex, and in the right hemisphere, in the precentral and paracentral cortex compared to MRI findings of CS (Fig. 1). The ROI analysis revealed significant cortical thinning in right (*p* = 0.019) and left (*p* = 0.016) precentral gyrus. Similarly, TBSS analysis revealed in ALS-EDA a reduction FA of bilateral CST from the cortex to the cerebral peduncles, more relevant in the left hemisphere (Fig. 2). Probabilistic tractography analysis confirmed the significant reduction of FA in bilateral CST (right CST: *p* = 0.035, left CST *p* = 0.002) in comparison to CS (Table 2).

**Table 2:** Cross sectional MRI measures in study populations

	ALS-EDA at T0 n.13	CS n.12	*p*	ALS_EDA at T6 n.11	ALS-RIL n.11	*p*
ROI analysis						
CT of the right precentral gyrus (mean ± SD) (median; range) (mm)	2.35 ± 0.18 2.35; 1.90–2.61	2.50 ± 0.15 2.51; 2.20–2.73	0.019	2.23 ± 0.24 2.22; 1.74–2.57	2.37 ± 0.16 2.41; 2.04–2.53	n.s
ROI analysis						
CT of the left precentral gyrus (mean ± SD) (median; range) (mm)	2.34 ± 0.17 2.35; 2.04–2.62	2.52 ± 0.14 2.51; 2.28–2.80	0.016	2.23 ± 0.27 2.24; 1.78–2.64	2.36 ± 0.10 2.36; 2.16–2.55	n.s
FA average Right CST (mean ± SD) (median; range)	0.47 ± 0.01 0.47; 0.44–0.49	0.49 ± 0.01 0.48; 0.47–0.51	0.035	0.46 ± 0.02 0.46; 0.43–0.50	0.44 ± 0.03 0.44; 0.41–0.49	n.s
FA average Left CST (mean ± SD) (median; range)	0.47 ± 0.02 0.48; 0.44–0.50	0.5 ± 0.02 0.49; 0.46–0.53	0.002	0.46 ± 0.03 0.46; 0.40–0.49	0.45 ± 0.02 0.46; 0.41–0.47	n.s

Mann–Whitney *U* test

*ALS- EDA* ALS patients taking edaravone in add on to riluzole, *CS* control subjects, *ALS-RIL* ALS patients taking riluzole, *CT* cortical thickness, *FA* fractional anisotropy, *CST* corticospinal tract

The comparison between ALS-EDA at T6 and ALS-RIL did not show differences in cortical thickness and FA of the CST bilaterally (Table 2).

### Longitudinal MRI analysis

**Fig. 3:**
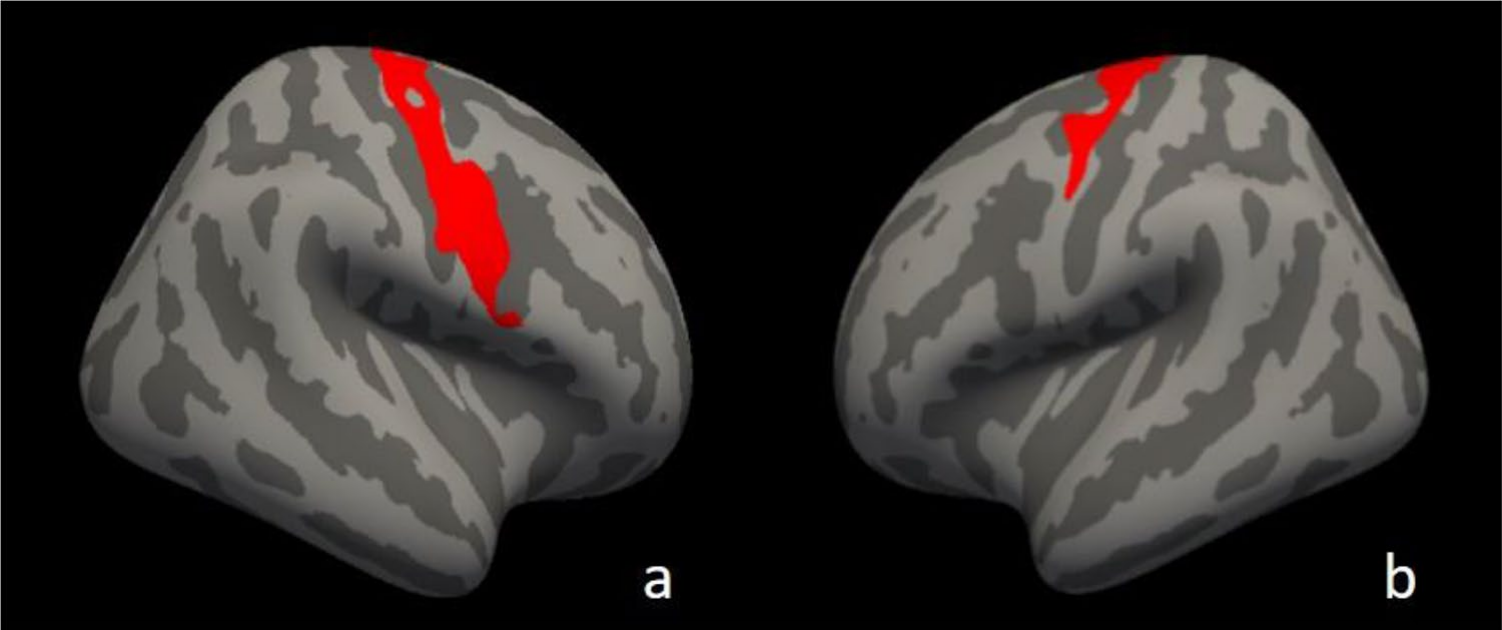
Symmetrized percent change maps of ALS-EDA patients after 6 months of treatment. Longitudinal analysis revealed cortical thinning in the bilateral precentral cortex in ALS-EDA after 6 months of treatment with edaravone in add on to riluzole. Coloured areas represented the significance maps after cluster-wise correction with a two-tailed permutation simulation in right hemisphere (**a**), and in left hemisphere (**b**)

**Fig. 4:**
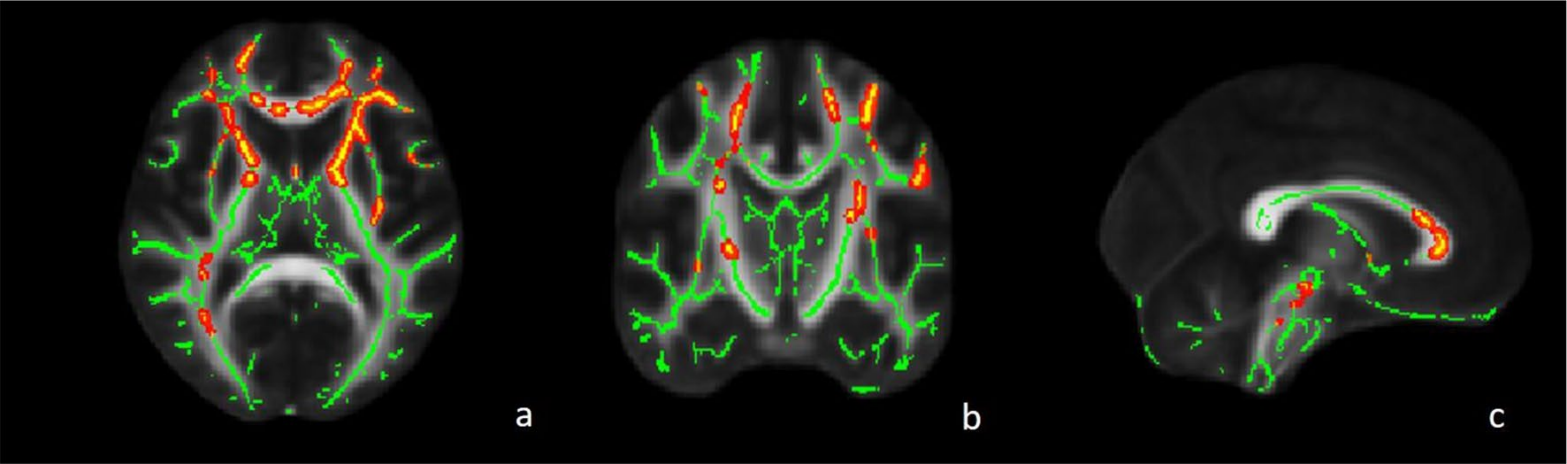
Longitudinal TBSS analysis of ALS-EDA patients after 6-months of follow-up. Areas with reduced FA are shown in yellow–red in anterior commissure, arcuate fasciculus, rostrum of corpus callosum and bilateral CST of ALS-EDA at T6. Regions of significant differences are overlaid on axial (**a**), coronal (**b**) and sagittal (**c**) of the MNI152 1 mm template

When compared to ALS-EDA at T6 vs T0, SPC of CT revealed one significant cluster in the bilateral precentral cortex with predominant impairment in the right hemisphere (cluster size: left: 1659.46 mm^2^, right: 2774.77 mm^2^) (Fig. 3). WM analysis showed a significant widespread reduction of FA in several white matter tracts, in particular, in anterior commissure, arcuate fasciculus, rostrum of corpus callosum and bilateral corticospinal pathways (Fig. 4). After ROI analysis, a significant reduction of FA values was found bilaterally in the CST (left hemisphere, *p* = 0.033; and right hemisphere, *p* = 0.041).

## Discussion

MRI generated measures have been proposed as potential diagnostic, prognostic biomarkers of ALS, nevertheless, to date, there are no published studies on MRI measures as an indicator of the effectiveness of drugs used in the disease. In this pioneering study, we have tried to investigate baseline and six-month MRI changes in real life on newly diagnosed ALS patients treated with edaravone, using CT and DTI analysis.

At baseline, the comparison between ALS patients and control subjects showed the typical pattern of ALS pathology in CT of both primary motor areas and in FA of both corticospinal. Almost all the studies carried out with CT analysis showed a predominant thinning of the precentral gyrus of ALS patients, confirming that the primary motor cortex is a distinctive site of microstructural alterations that characterizes the disease [28]. Likewise, DTI studies found alterations of the corticospinal tracts, which appears rapidly, already in the early stages of the disease, due to damage to the neurolemma or the myelin sheath [29]. The homogeneity of the results of the cross-sectional studies suggested the use of MRI as an optimal biomarker of ALS pathology [30].

Our objective was to evaluate the effect of edaravone on MRI measures and, for this purpose, MRI examination has been repeated after six months which, although short, a proportionate time considering the rapid course of the disease. Comparing baseline with follow-up MRI metrics, the most relevant results are that, at six months, CT analysis showed further cortical thinning in the motor areas. Similarly, TBSS showed a greater involvement of corticospinal tracts, but also a widespread involvement of other strategical traits of the ALS pathology [31]. Differently from MRI cross-sectional literature data, longitudinal studies did not report univocal results [32–34]. It seems that the discrepancy of the data reflect the heterogeneity of the progression of the disease [10]. To evaluate structural changes during the course of the disease, it is necessary to select clinically homogeneous groups. Our study group, although small, has the advantage of being representative of an early stage of disease, due to the restrictive criteria of the enrolment.

The widespread involvement of white matter tracts that we found at T6 includes the anterior commissure, the rostrum of the corpus callosum and the nearest frontal tracts. This pattern could be a confirmation of the prion-like hypothesis of disease progression, through the bi-hemispheric connections [35]. In this regard, an important emphasis has been given to the role of the corpus callosum in ALS. Histopathological evidences and more recent DTI and voxel-based morphometry studies have all confirmed a degeneration of the corpus callosum in ALS [36–39].

In the planning phase of the study, we ran into the problem of defining the ALS group to compare with ALS-EDA, because, for ethical reasons, we could not avoid administering edaravone to patients who could benefit from it. To overcome this obstacle, we used PSM to extract from our ALS database a group of matched patients taking only riluzole to compare with ALS-EDA group. To date, several studies used this method to obtain a matched sample with similar MRI measures [40–42]. Our results did not show evidence of a difference between edaravone and riluzole. This evidence is in agreement with clinical data by the Italian EDARAVALS Study Group, which reported no benefit of this drug in terms of disease progression and respiratory function [9].

One limit of our single centre real life study is the small sample size. However, as reported in sample size calculation section, the number of recruited patients is widely sufficient to detect difference in CT of precentral gyrus as previously reported in literature. To our knowledge, no data of a minimal detectable change of the FA of CST are available in two compared cross-sectionally ALS groups of patients. One study conducted in stroke patients reported 0.015 as the minimal detectable change in FA of CST, using the probabilistic tract approach [43]. The minimal difference found in our study is still adequate in our opinion, considering that this value was obtained in a non-easily reproducible setting with a widespread disease.

Another possible limit is the short time of follow-up; however, we feel that these did not bias our results, indicating that patients treated with edaravone showed progression of damage in the motor cortex and several WM tracts.

In our opinion, this study might provide a methodical approach that could be applied in other pharmacological clinical studies in ALS disease.

## Data Availability

The data that support the findings of this study are available from the corresponding author, [ILS], upon reasonable request**.**
